# Embryonal tumor with multilayered rosettes: a report of a rare case

**DOI:** 10.1259/bjrcr.20210216

**Published:** 2022-04-01

**Authors:** Sultan A. Alshoabi, Ali M. Abdu, Hisham M. Alkhalidi, Miral D Jhaveri, Abdullgabbar Hamid

**Affiliations:** 1 College of Applied Medical Sciences, Taibah University, , KSA, Almadinah, Kingdom of Saudi Arabia; 2 Radiology Unit, King Saud University Medical City, Riyadh, Kingdom of Saudi Arabia; 3 Department of Pathology, College of Medicine, King Saud University, Riyadh, Kingdom of Saudi Arabia; 4 Department of Radiology, Rush University Medical Center, Chicago, Illinois, USA

## Abstract

The authors report a case of an embryonal tumor with multilayered rosettes (ETMR) in an 18-month-old female infant who presented with gait imbalance and progressive left-sided weakness for 2 months. ETMR is a rare small round blue cell aggressive tumor of the central nervous system characterized by the amplification of the C19MC region on chromosome 19 (Chr19q13.42). This report in detail the clinical–radiologic and histopathological workup and diagnosis. Because ETMRs are newly described rare pediatric central nervous system tumors with only a few reported cases, we aim to document this typical case to add to the existing data on these tumors.

## Introduction

Embryonal tumor with multilayered rosettes (ETMR), C19MC-altered, is a rare malignant pediatric brain tumor recently included in the 2016 World Health Organization (WHO) update classification of central nervous system (CNS) neoplasms and is also present in the latest version of WHO classification of CNS tumors 2021.^1^ This aggressive tumor has a hallmark cytogenetic abnormality with amplification of the C19MC region on chromosome 19 (Chr19q13.42).^1,2^ ETMR usually affects children before their fourth birthday and can occur anywhere in the brain with highly aggressive behavior and poor prognosis. Morphologically, it is characterized by multilayered rosettes and belongs histologically to WHO Grade VI neoplasms.^2,3^ Because ETMRs are newly described rare pediatric CNS tumors with only a few reported cases, we aim to document this typical presentation to add to the existing data on these tumors.

## Case presentation

An 18-month-old female initially presented with right eye pain and outward deviation. Evaluation by an ophthalmologist led to the diagnosis of conjunctivitis. Over the next 2 months, the patient developed gait imbalance, frequent falls, and left-sided weakness with drooling of saliva, leading to a referral to a neurologist.

### On examination

The patient was conscious and alert with normal tone, normal reflexes, and no signs of increased intracranial pressure. The patient had an ataxic gait, drooling of saliva on the left, and decreased motor strength on the left.

### Vitals signs and measurements

On admission, the temperature was 36.9°C, respiratory rate was 28 cycles/min, blood pressure was 86/56 mmHg, SpO2 was 98%, height was 83 cm, weight was 17.5 kg, and 25.4 body mass index.

**Figure 1.**
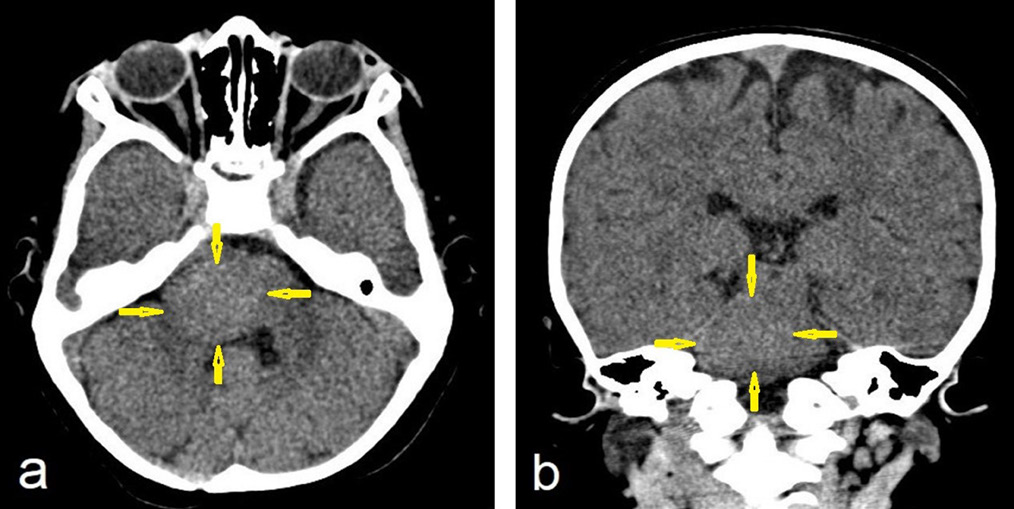
Non-enhanced CT of the brain (a) axial and (b) coronal images show an isodense mass (arrows) in the right side of the pons extended into the right middle cerebellar peduncle with mass effect and distortion of the fourth ventricle.

A head CT was performed, which demonstrated an isodense to hyperdense mass with a well-defined margin involving the right lateral aspect of the pons, middle cerebellar peduncle, and the cerebellopontine angle. In addition, there was mass effect with partial effacement on the fourth ventricle, left deviation of the pons with no herniation or hydrocephalus (Figure 1a & b).

**Figure 2.**
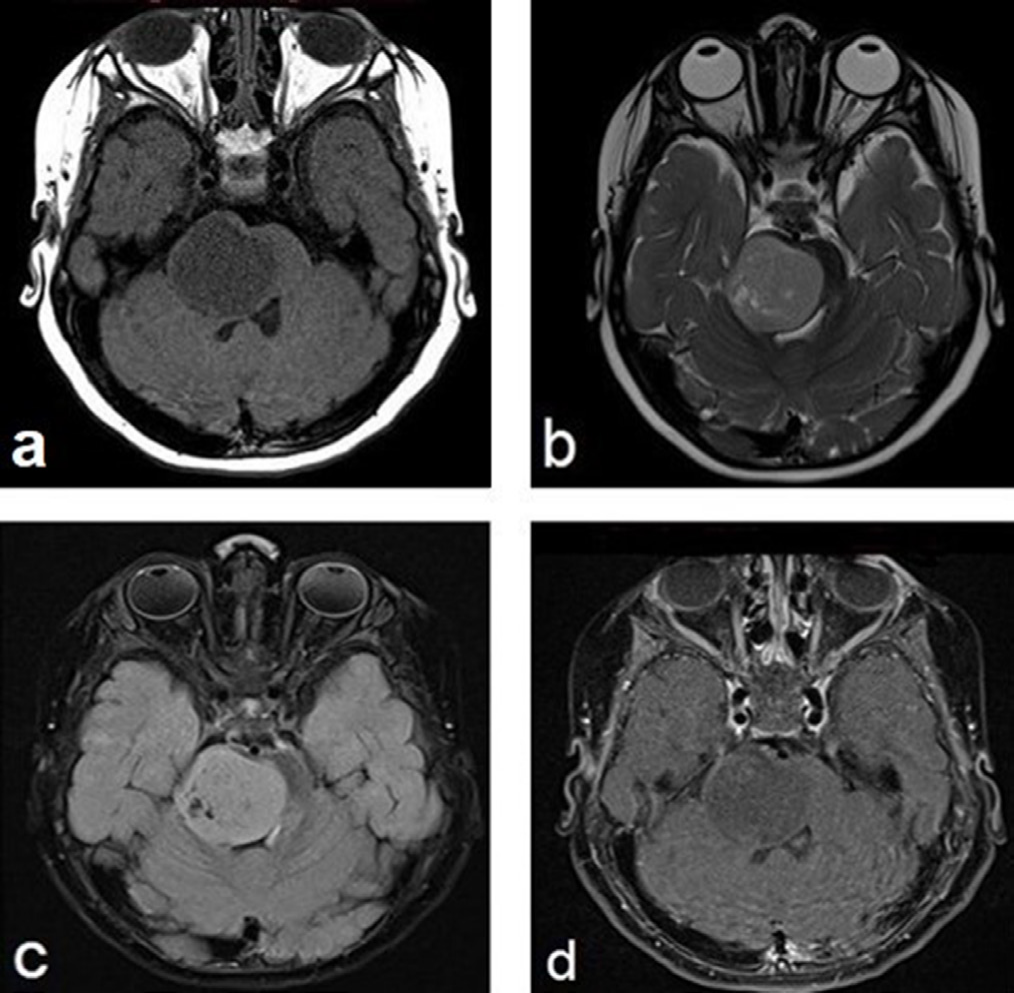
Brain MRI images demonstrate a right-sided pontine mass extending to the right middle cerebellar peduncle with mass effect on the fourth ventricle. The tumor is hypointense on *T*_1_WI (**a**) and hyperintense on both *T*_2_WI (**b**) and FLAIR (**c**) with minimal enhancement after contrast administration (**d**). FLAIR, fluid attenuation inversion recovery; *T*_1_WI, *T*_1_ weighted imaging; *T*_2_WI, *T*_2_ weighted imaging;

**Figure 3.**
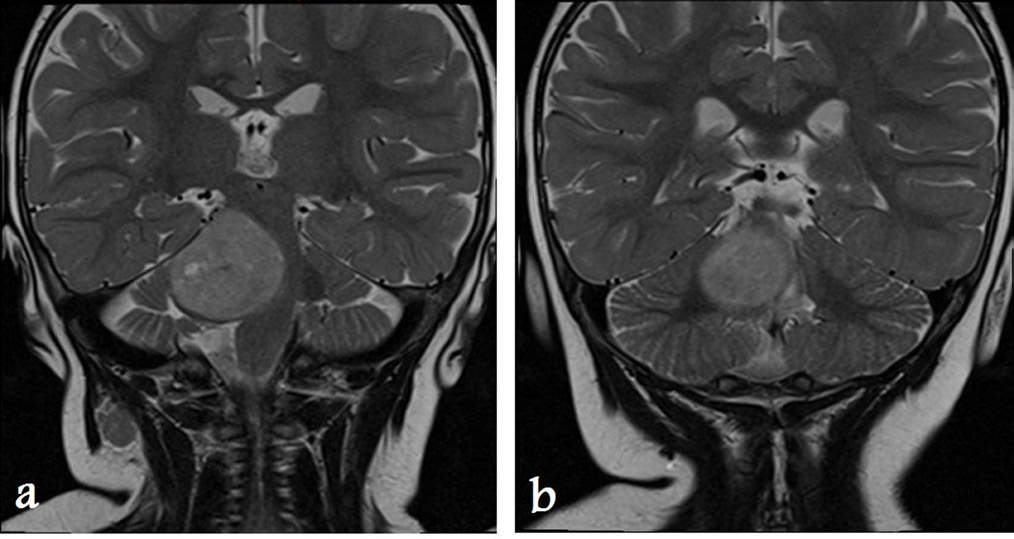
Coronal T2 MRI images of the same hyperintense lesion on the right side of the pons with mass effect on the brain stem and fourth ventricle.

**Figure 4.**
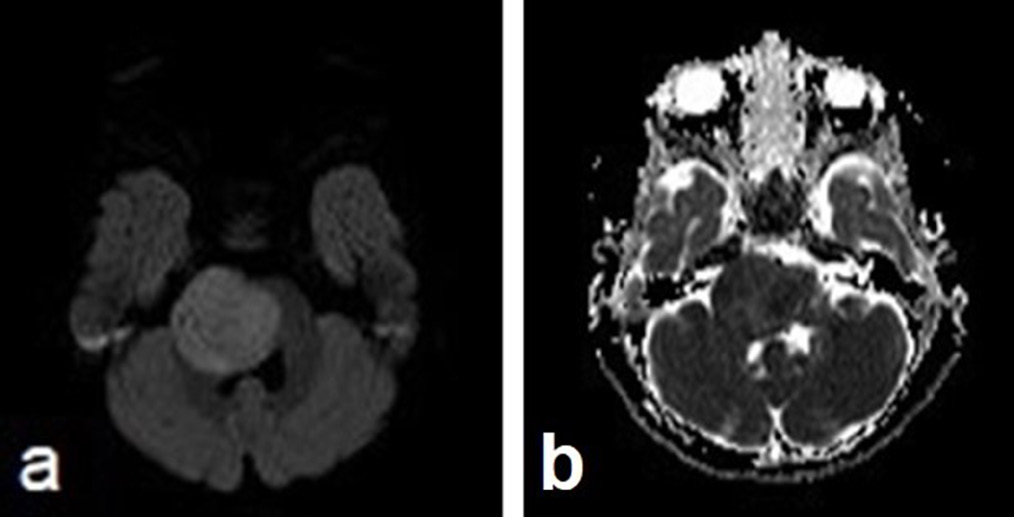
Axial MRI images show mild restricted diffusion. The tumor is mildly hyperintense on the diffusion-weighted image (**a**) and hypointense on the ADC map (**b**). ADC, apparent diffusion coefficient.

**Figure 5.**
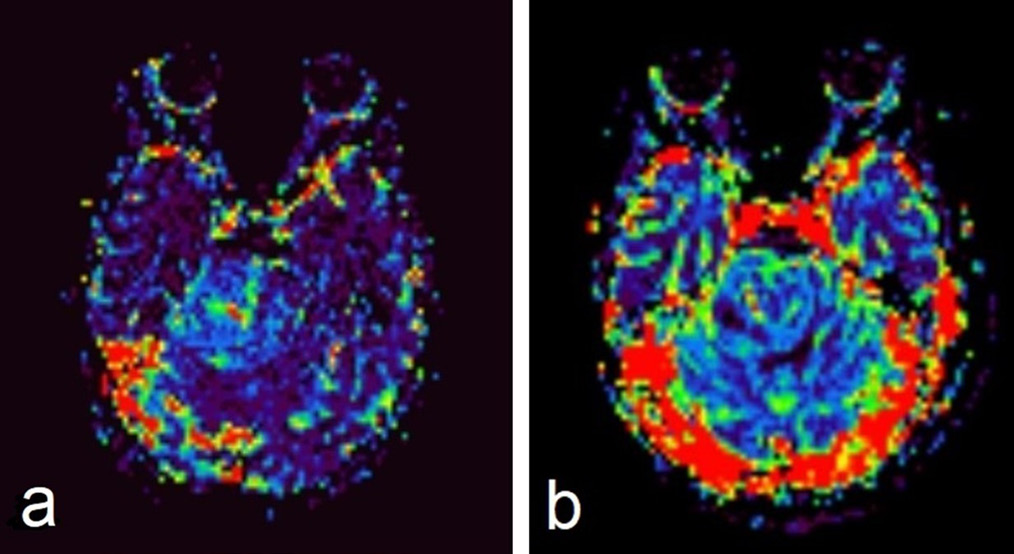
MR perfusion (rCBV) shows high relative cerebral blood volume in the pontine mass. rCBV, relative cerebral blood volume.

Subsequently, MRI demonstrated a right pontine and cerebellopontine angle mass with low signal intensity on *T*_1_ weighted images (*T*_1_WI), (Figure 2a), mildly high signal on *T*_2_ weighted images (*T*_2_WI), (Figures 2b and 3), isointense to mild high signal on fluid attenuation inversion recovery-weighted images (FLAIR), (Figure 2c), and minimal enhancement after contrast administration (Figure 2d). Diffusion-weighted image (DWI, Figure 4a) and apparent diffusion coefficient (ADC, Figure 4b) map showed mild restricted diffusion. MR perfusion showed mildly elevated relative cerebral blood volume (rCBV, Figure 5). Based on the imaging appearance, a diagnosis of a brainstem embryonal tumor was entertained.

**Pre-operative** MRI of the whole spine did not demonstrate any drop metastasis. CSF analysis was also negative.

Under general anesthesia, tumor debulking surgery was performed via a right suboccipital craniotomy. The specimen was sent for frozen and permanent pathology.

**Figure 6.**
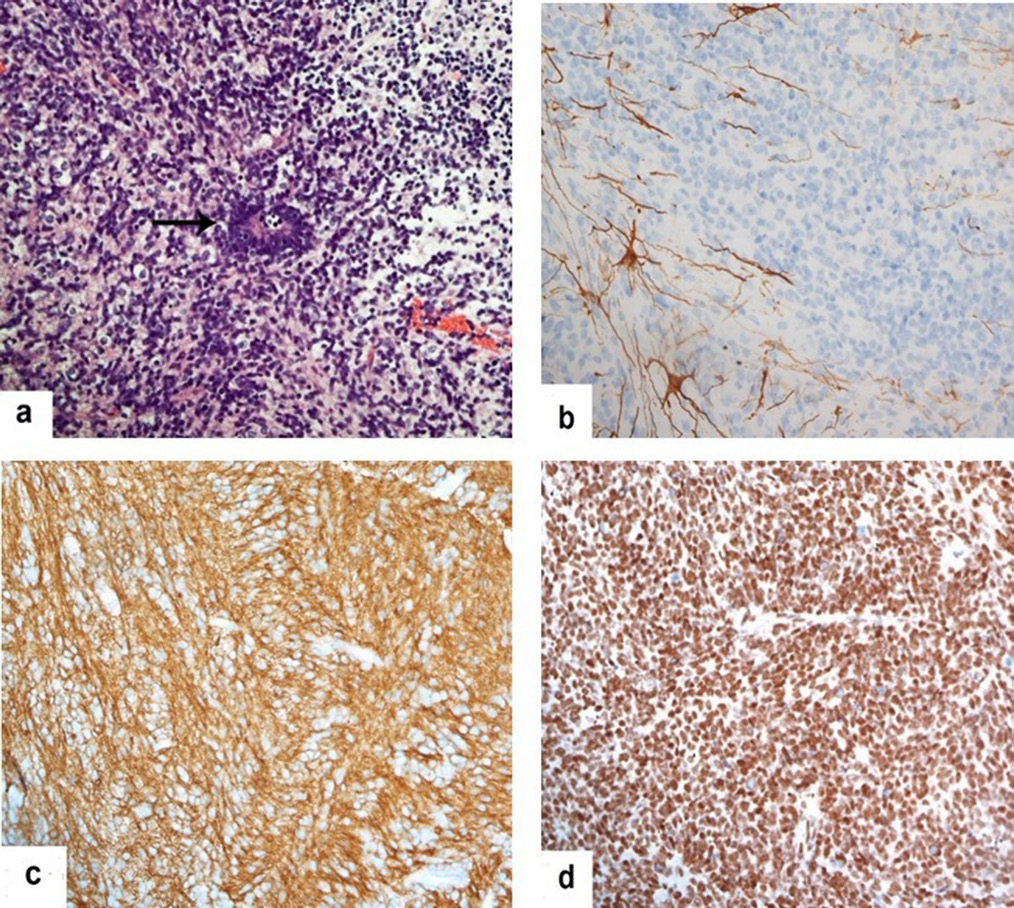
Selected images of histopathology examination show (a) the tumor consists of small round blue cells with scanty cytoplasm and indistinct cell borders. It exhibits dense cellular areas (left half of the field) and paucicellular areas (right half of the field). Multilayered rosettes (arrow) are scattered (H&E, X200), (**b**) the tumor is negative for GFAP. Scattered reactive astrocytes are noted focally in the background (GFAP, X200), (**c**) the tumor cells are diffusely positive for CD56; in keeping with the neural nature of the neoplasm (CD56, X200), and (d) INI1 shows normal nuclear reactivity, excluding the possibility of Atypical Teratoid Rhabdoid Tumors (INI-1, X200).

**The histological** features were classically associated with *C19MC* alteration(Figure 6). These features included multilayered Rosettes that consisted of pseudostratified neuroepithelium with a central and round empty lumen. The nuclei of the rosette-forming cells tend to be pushed away from the lumen towards the outer cell border. The background was dominated by sheets and clusters of poorly differentiated cells with a high nuclear-to-cytoplasmic ratio, a paucity of a neutrophil matrix, and ganglion cell elements. Immunohistochemistry studies showed that the tumor cells were positive for CD56 and synaptophysin. They were negative for SMA, EMA, chromogranin, and NeuN. GFAP highlighted reactive astrocytes and INI-1 was intact. P53 showed focal reactivity in the tumor cells. The diagnostic genetic marker for the tumor is an upregulation of the *C19MC* gene, which unfortunately was not performed. The differential diagnoses were with the group of embryonal tumors of the CNS *[e.g.* embryonal tumor, NOS (not otherwise specified)].

Unfortunately, genetic amplification was not available at that time. Nevertheless, two independent histopathologists confirmed the morphological appearance consistent with the diagnosis of an ETMR, NOS (WHO Grade IV).

## Discussion

In 2016, the WHO classification of CNS tumors added molecular parameters to histology to define CNS tumors and incorporates new entities. ETMR includes the previously known embryonal tumors with abundant neuropil and true rosettes (ETANTR) that also referred to ETMR, ependymoblastoma, and, in some patients, medulloepithelioma. The presence of *C19MC* amplification results in a diagnosis of ETMR*, C19MC-altered*.^4^^,^ While its absence results in the diagnosis of ETMR- NOS. In our report, we document a new case of ETMR in a female infant who initially presented with right eye pain and exophthalmos, then developing gait imbalance and left-sided weakness. These findings were consistent with a posterior fossa lesion. As with other intracranial masses, the most common clinical features of ETMRs are symptoms and signs of increase intracranial pressure and focal neurological deficits. However, clinical features of ETMRs vary according to the tumor’s site and size, which may occur either above or below the tentorium in the cerebral hemispheres or in the cerebellum, brain stem, or even in the spinal cord.^3,5^ In our study, the tumor was in the right side of the pons and middle cerebellar peduncle with mass effect on the fourth ventricle.

On MRI, our patient, the mass was low signal intensity on *T*_1_WIs with minimal heterogeneous enhancement after contrast administration, high signal intensity on *T*_2_WIs with no surrounding high signal intensity edema on FLAIR. It also demonstrated restricted diffusion on DWIs with low ADC value and slightly elevated rCBV on MR perfusion. These findings are consistent with previous studies that reported that ETMRs were frequently hypointense on *T*_1_WIs, hyperintense on *T*_2_WIs, with weak and heterogeneous enhancement, restricted diffusion, and high cerebral blood volume (CBV).^1,6^ The high rCBV at MR perfusion could be secondary to increased vascular permeability and increased vascular endothelial hyperplasia.^7^ Some authors reported more elevated choline at MRS in this type of tumor than in malignant gliomas, reflecting higher cellularity and mitotic activity in ETMRs.^7^

Multiple studies reviewed the immunohistochemical and genetic features of ETMRs. On this patient, the histological features were classically associated with C19MC alteration. Dangouloff-Ros et al reported that ETMRs could be suspected histopathologically by LIN28A-positive immunostaining, but this should be confirmed with molecular confirmation of C19MC alteration.^1^ In our patient, histopathology examination showed scattered multilayered rosettes with scattered reactive astrocytes. Govindan et al reported that the hallmark for histological diagnosis of ETMR is the presence of ependymoblastic rosettes and small undifferentiated cells.^8^ The immunohistochemistry in our patient showed that the tumor cells were positive for CD56 and synaptophysin. Wang et al reported that synaptophysin neuronal marker was positive in the differentiated areas of ETMRs, neurons with positive NeuN were found occasionally, and Ki-67 proliferative index was observed in up to 40–80% of rosettes and undifferentiated cells. Wang et al also reported that Nestin was a positive immunoreactive neural stem cell marker in ETMRs.^9^ Shih et al reported that amplification of C19MC and loss of INI-1 expression is necessary to define ETMRs.^7^ This is in line with our patient, which showed intact INI-1. The histological features in our patient are consistent with C19MC alteration.

ETMRs are highly aggressive brain tumors with amplification of the *C19MC* locus at chromosome 19q13.41 miRNA cluster and enrichment of the LIN28A pluripotency factor. However, the pathogenesis and molecular landscape of ETMRs remains idiopathic.^10^ Wang et al reported that 19q13.42 amplifications were detected in 30% of ETMRs.^9^

ETMRs have a poor prognosis even after total resection surgery due to CSF dissemination and metastasis. Typical treatment is trimodal with safe resection, adjuvant chemotherapy, and craniospinal irradiation except in children under 3 years old.^7^ Bailey et al reported that ^131^I-omburtamab is safe with a favorable therapeutic index in ETMRs when used following surgery and chemoradiation therapy.^11^

### Limitations

This case report is limited because the genetic studies were unavailable, and the diagnosis was solely on the typical MRI features and histopathology results.

## Conclusion

ETMR is a rare, highly aggressive pediatric age group tumor that can occur throughout the brain with variable clinical features based on location. ETMRs demonstrate characteristic MRI features, and multilayered rosettes should be present on histopathology to diagnose it. C19MC amplification is required to diagnose C19MC altered type. However, if this amplification is not confirmed, ETMR-NOS should be the diagnosis.

## Learning points

- Awareness of the new WHO classification of the brain tumors among the radiologists should be raised.
- The new tumor classes are highly dependent on the genetic features.
- ETMR are highly aggressive tumors with typical CT and MRI features.

Informed consent in writing was obtained from the child’s father for reporting and publishing the clinical records.
